# Isoquercetin and inulin synergistically modulate the gut microbiome to prevent development of the metabolic syndrome in mice fed a high fat diet

**DOI:** 10.1038/s41598-018-28521-8

**Published:** 2018-07-04

**Authors:** Si Tan, Jose A. Caparros-Martin, Vance B. Matthews, Henrietta Koch, Fergal O’Gara, Kevin D. Croft, Natalie C. Ward

**Affiliations:** 1 Life Science and Technology Institute, Yangtze Normal University, Chongqing, China; 2 School of Biomedical Sciences, University of Western Australia, Perth, Australia; 3 School of Biomedical Sciences & Curtin Health Innovation Research Institute, Curtin University, Perth, Australia; 4 Biomerit Research Centre, School of Microbiology, National University of Ireland, Cork, Ireland; 5 Medical School, University of Western Australia, Perth, Australia

## Abstract

Dietary fibre positively influences gut microbiome composition, enhancing the metabolism of dietary flavonoids to produce bioactive metabolites. These synergistic activities facilitate the beneficial effects of dietary flavonoids on cardiometabolic health parameters. The aims of this study were to investigate whether isoquercetin (a major dietary flavonoid) and inulin (soluble fibre), either alone or in combination could improve features of the metabolic syndrome. Following a 1 week acclimatization, male C57BL6 mice (6–8 weeks) were randomly assigned to; (i) normal chow diet (n = 10), (ii) high fat (HF) diet (n = 10), (iii) HF diet + 0.05% isoquercetin (n = 10), (iv) HF diet + 5% inulin, or (v) HF diet + 0.05% isoquercetin + 5% inulin (n = 10). Body weight and food intake were measured weekly. At 12 weeks, glucose and insulin tolerance tests were performed, and blood, faecal samples, liver, skeletal muscle and adipose tissue were collected. At 12 weeks, mice on the HF diet had significantly elevated body weights as well as impaired glucose tolerance and insulin sensitivity compared to the normal chow mice. Supplementation with either isoquercetin or inulin had no effect, however mice receiving the combination had attenuated weight gain, improved glucose tolerance and insulin sensitivity, reduced hepatic lipid accumulation, adipocyte hypertrophy, circulating leptin and adipose FGF21 levels, compared to mice receiving the HF diet. Additionally, mice on the combination diet had improvements in the composition and functionality of their gut microbiome as well as production of short chain fatty acids. In conclusion, long-term supplementation with the dietary flavonoid isoquercetin and the soluble fibre inulin can attenuate development of the metabolic syndrome in mice fed a high fat diet. This protective effect appears to be mediated, in part, through beneficial changes to the microbiome.

## Introduction

The metabolic syndrome is defined as a series of risk factors that commonly cluster together, increasing the risk of both cardiovascular disease (CVD) and type 2 diabetes (T2DM)^1,2^. Six contributing components have been identified, including abdominal obesity, dyslipidemia, hypertension, insulin resistance, pro-inflammatory state and pro-thrombotic state^1^. Poor or suboptimal nutrition is a leading risk factor for death and disability worldwide^3^ with a recent population study suggesting that a diet low in fruits and vegetables is a major behavioural risk factor for non-communicable diseases after hypertension and smoking^4^. Diet has been shown to influence a range of cardiometabolic risk factors, including glucose-insulin homeostasis, blood pressure, lipid profile, metabolic expenditure, adiposity, hepatic function and the gut microbiome^3,5^. Importantly, diet-related diseases such as CVD and T2DM remain major global health burdens^3^.

The microbiome plays an important role in our physiology, immune system development, digestion and overall health^6^. Most of the microbiota live in the gut, which accounts for >5 million different genes and >1000 different species, all of which belong to a small number of phyla^6,7^. Despite the enormous diversity and complexity of the microbiota, it can be divided into enterotypes or clusters of living organisms based on their bacterial ecosystem^6,8^. Microbial richness and the metabolic capacity of the bacterial community is considered an indicator of health status, with dysbiosis associated with obesity, immune and inflammatory diseases^6^, and metabolic diseases such as CVD and T2DM^7,9^. While extremely dynamic, the microbiota composition and structure can be influenced by a number of factors including diet and individual dietary components^6,10,11^.

Fruits and vegetables are comprised of a number of functional compounds, including; flavonoids and fibre, which are thought to contribute to the beneficial effects of a diet rich in fruits and vegetables^12^. Plant-derived flavonoids have been associated with beneficial effects on a range of metabolic processes^3,5^. Dietary flavonoids predominantly exist conjugated to other moieties, particularly sugars, and the cleavage of these moieties and subsequent metabolism is important for both absorption and biological activity of the parent compound and its metabolites^5^. In addition to cleavage and hydrolysis, colonic microbiota also play a role in the metabolism of flavonoids, which affects their absorption and transit time in the small and large intestine and circulatory time in the plasma^5,11–13^. Inulin is a soluble dietary fibre composed of a group of naturally occurring polysaccharides, which are not digested in the intestine but instead, fermented into short-chain fatty acids (SCFAs). These SCFAs have been associated with several key metabolic processes, including adiposity, food intake, lipid metabolism, glucose homeostasis and insulin resistance^14,15^. In addition to these prebiotic characteristics, inulin also plays a role in regulating hepatic lipid metabolism and improving lipid profiles^16,17^.

A gut microbiome composition that promotes the metabolism and absorption of dietary flavonoids may play a crucial role in promoting their beneficial effects. Therefore, the aims of this study were to investigate the long-term effects of flavonoid and fibre supplementation, alone and in combination, on gut microbiome composition and development of the metabolic syndrome in mice fed a high fat diet. Isoquercetin (ISO), a glucoside of quercetin (a common dietary flavonoid) that is commercially available in pure form, and inulin (INU), a non-digestible polysaccharide, were used as sources of flavonoid and fibre, which were added to a high fat (HF) diet and fed to male C57BL6 mice for a period of 12 weeks.

## Methods

### Animal study

Male C57BL6 mice (6–8 weeks) were purchased from the Animal Resource Centre (Perth, Australia) and maintained at 23 ± 2 °C under a 12 hour light-dark cycle. Following a week of acclimatisation, the mice were randomly divided into one of five groups (n = 10, 5 mice/cage); (i) normal chow diet (Chow), (ii) high fat diet (HF), (iii) HF diet + 0.05% isoquercetin (wt/wt) (ISO), (iv) HF diet + 5% inulin (wt/wt) (INU), and (v) HF diet + 0.05% isoquercetin + 5% inulin (ISO + INU). The normal chow diet was commercial rodent chow consisting of 4.8% wt/wt fat, 19% protein, 59.9% carbohydrate and 5.2% crude fibre, with digestible energy 14.2MJ/Kg. The HF diet contained 21% wt/wt fat (clarified butter) and 0.15% cholesterol, 23% protein, 49% carbohydrate (34% sucrose) and 4.7% crude fibre, with digestible energy 19.4MJ/Kg. Mice were allowed ad libitum access to water and food and all diets were prepared by Specialty Feeds (Glenn Forrest, Australia). The mice were maintained on their respective diets for 12 weeks. The doses of isoquercetin and inulin corresponded to 3–4 apples/day and the recommended daily intake of fibre (25–30 g/day), based on mice consuming ~5 g food/day and when converted to human doses^18,19^. Body weight and food intake were measured weekly. The study was approved by the Royal Perth Hospital Animal Ethics Committee (R532/16–17). All animal experiments were compliant with the National Health and Medical Research Council (NHMRC) guidelines for the Care and Use of Laboratory Animals in Australia.

### Glucose and insulin tolerance tests

Intraperitoneal glucose tolerance test (IPGTT) and insulin tolerance test (IPITT) were performed on fasting (5 hr) mice at weeks 11 and 12 respectively. To measure blood glucose levels, blood samples were taken from the tail of fasting (5 hr) mice before (t = 0 min) and at subsequent time intervals of t = 15, 30, 45, 60, 90 and 120 min following intraperitoneal administration of 1 g glucose/kg and 0.5U insulin/kg body weight for IPGTT and IPITT respectively. Blood glucose levels were measured using Accu-Chek Performa Strips and Glucometer (Roche Diagnostics, Australia). The area under the curves (AUCs) for the IPGTTs and IPITTs were calculated using the trapezoidal method.

### Serum biochemistry

Fasted (5 hr) mice were anaesthetised with methoxyflurane at week 12 and blood samples obtained via cardiac puncture. Serum was separated via centrifugation (3000 rpm, 4 °C, 10 mins) and stored at −80 °C until analysis. Serum total cholesterol (TC), low-density liprotein (LDL), triglycerides (TG) and high-density lipoprotein (HDL) were analysed by PathWest Laboratories (Fiona Stanley Hospital, Perth). Serum insulin was measured using a commercially available enzyme-linked immunosorbent (ELISA) kit (Millipore, USA) and serum leptin using a commercially available ELISA kit (R&D Systems, UK).

### Tissue histology and pathology

Fasted (5 hr) mice were anesthetised with methoxyflurane at week 12 and liver and white adipose tissue were collected and fixed in 4% formaldehyde overnight before being incubated in 50% ethanol and embedded in paraffin. The tissues were then cut into 4 *μ*m sections and stained with hematoxylin and eosin (Cell Central, UWA) before being visualized and photographed using a Nikon Eclipse TS100 microscope. Additional liver, white adipose tissue and skeletal muscle was collected and snap frozen in liquid nitrogen and stored at −80 °C until analysis. Total fat content of the liver was determined following Folch extraction as previously described^20^. Hepatic and skeletal muscle tissue protein expression of GLUT4 (Cell Signaling, USA), insulin receptor (Cell Signaling, USA), heme-oxygenase-1 (Hmox-1, Enzo), AMPK (Cell Signaling, USA), ACC (Cell Signaling, USA) and Akt (Cell Signaling, USA) were determined using western blot and normalised to actin (Sigma, USA), as previously described^21^. Adipose tissue levels of fibroblast growth factor-21 (FGF21) were quantitated using an ELISA kit (R&D Systems).

### Faecal and serum short chain fatty acid (SCFA) analysis

In addition to the fasting serum sample, faecal pellets were collected at week 12 and stored at −80 °C until analysis. Serum and faecal SCFA concentrations were analysed using gas chromatography mass spectrometry as previously described^22^.

### Gut microbiome analysis

DNA was isolated from collected faecal samples using a QIAamp Fast DNA Mini Kit (QIAGEN), following manufacturer’s instructions. PCR amplification of the V3-V5 region of the hypervariable region of the *16 s rRNA* gene, library preparation and sequencing on a MiSeq desktop sequencer using V3 chemistry were carried out by a service provider (Eurofins Genomics). Demultiplexed reads were processed using minimum entropy decomposition^23,24^ to generate operational taxonomic unit (OTUs) clusters. Taxonomic assignment down to species level was performed using the QIIME package (version1.8.0)^25^. Analysis of the microbiome community was carried out using R (version 3.2.4). Raw microbiome sequencing data counts were prefiltered to remove OTUs with low counts and normalised by sum scaling normalisation. Resulting compositional data was transformed using Isometric Log Ratio as implemented in the function *pca* in the R package MixOmics^26^ before performing non-supervised multivariate analysis (PCA). For linear discriminant analysis, tab-delimited files were generated in R and LEfSe analysis^27^ computed at OTU level using Galaxy. Only discriminative features with logarithmic LDA score higher than 3 (absolute value) were retained and represented. Alpha values for Kruskal-Wallis and Wilcoxon test were less than 0.05. Prediction of the OTU-associated microbiome was carried out using PICRUSt^28^. Closed reference OTUs with 97% similarity were picked against the Greengenes database using MacQIIME. OTU number was normalised for *16 s rRNA* gene copy number and gene families predicted at level 3 KEGG orthology groups. To study the effect of the food supplements on KEGG pathway composition, the relative abundance of each KEGG family gene was calculated and then normalised to a total number of 25,000 genes.

### Statistical analysis

Statistical analysis was performed using the SPSS package (Version 21.0, Chicago USA) and R. All values are expressed as mean ± standard error (SEM). Data was analysed using repeated measures analysis of variance (ANOVA) and one-way ANOVA with Tukey HSD post-hoc analysis to determine differences between the groups. A p < 0.05 was determined to be of statistical significance. All data presented within the manuscript is available on request to the corresponding author.

## Results

### Isoquercetin and inulin synergistically protect against diet-induced obesity

**Figure 1:**
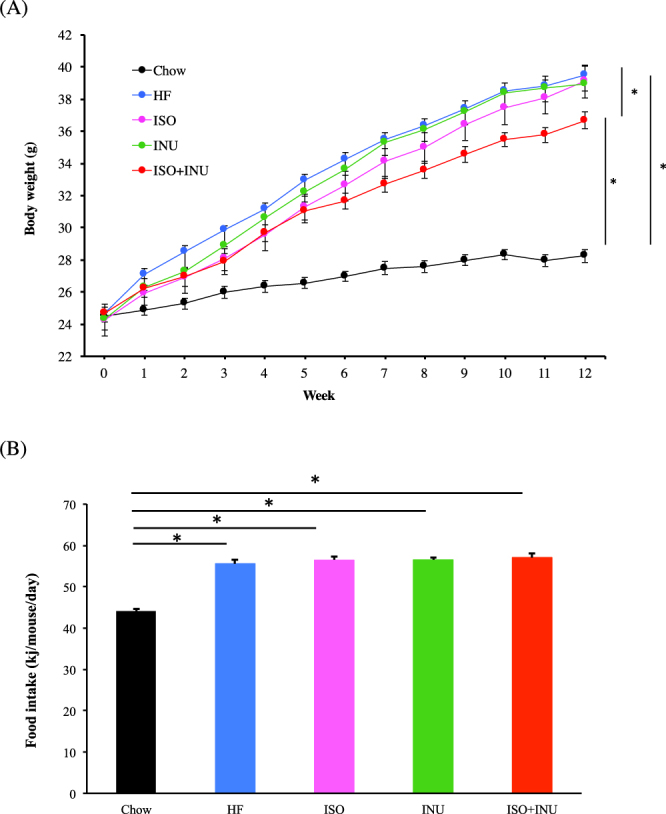
Supplementation with isoquercetin and inulin prevents weight gain despite no change in food intake in mice fed a high fat diet. (**A**) body weight and (**B**) average kilojoule intake in mice receiving the normal diet (Chow), high fat diet (HF) or HF supplemented with isoquercetin (ISO), inulin (INU) or the combination (ISO + INU) for 12 weeks. Mean ± SEM, ANOVA n = 10 per group, *p < 0.05.

Consumption of the HF diet for 12 weeks resulted in significantly greater weight gain compared to mice fed the normal chow diet. Supplementation of the HF diet with either isoquercetin or inulin alone had no effect on weight gain. In mice receiving the combination of isoquercetin and inulin, weight gain was significantly attenuated (Fig. 1A). Estimated food intake was significantly higher in the mice receiving the normal chow diet compared to those receiving the HF diets and this was not affected by the presence of isoquercetin or inulin, either alone or in combination. Overall kilojoule consumption was higher in the mice receiving the HF diets and this was not affected by the presence of either isoquercetin or inulin (Fig. 1B).

### Isoquercetin and inulin synergistically protect against diet-induced glucose intolerance and insulin resistance

**Figure 2:**
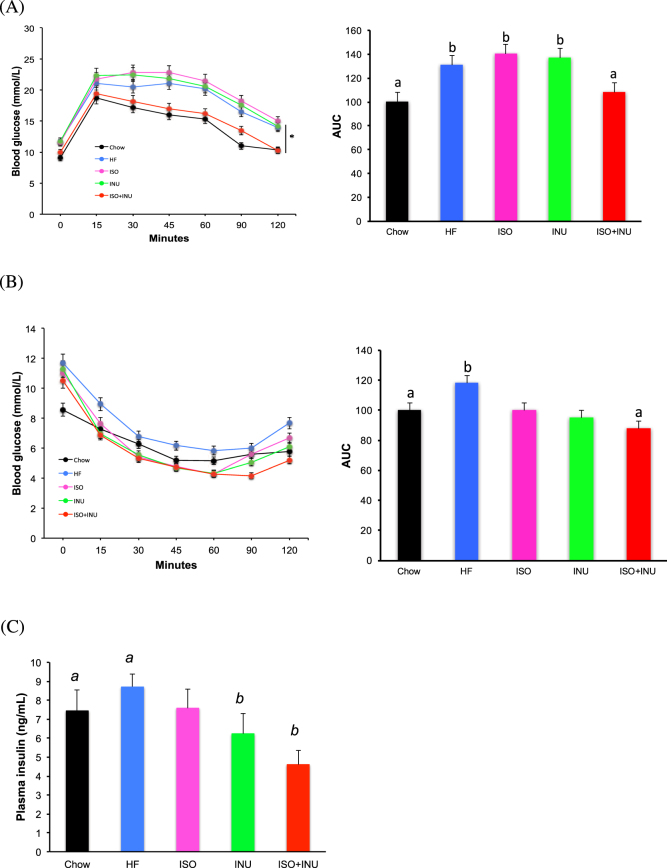
Supplementation with isoquercetin and inulin prevents the development of glucose intolerance and insulin resistance in mice fed a high fat diet. Fasting (**A**) glucose tolerance testing, (**B**) insulin tolerance testing and (**C**) circulating plasma insulin levels in mice receiving the normal diet (Chow), high fat diet (HF) or HF supplemented with isoquercetin (ISO), inulin (INU) or the combination (ISO + INU) for 11–12 weeks. Mean±SEM, repeated measures ANOVA n = 9–10 per group, *and different letters (a versus b) are statistically significant *p < 0.05.

Mice fed the HF diet for 12 weeks had significantly impaired glucose tolerance compared to the normal chow diet. Addition of either isoquercetin or inulin to the HF diet had no effect, however when given in combination, glucose tolerance was significantly improved and comparable to mice receiving the normal chow diet (Fig. 2A). In addition, the HF diet significantly increased insulin resistance compared to mice fed the normal chow diet. Again, addition of either isoquercetin or inulin to the HF diet had no effect on insulin resistance, except when they were added in combination (Fig. 2B). Hyperinsulinemia was evident in mice fed a HF diet and circulating levels of insulin were significantly decreased in mice fed a HF diet containing inulin alone or in combination with isoquercetin (Fig. 2C).

### Isoquercetin and inulin synergistically affect metabolic signalling pathways

**Figure 3:**
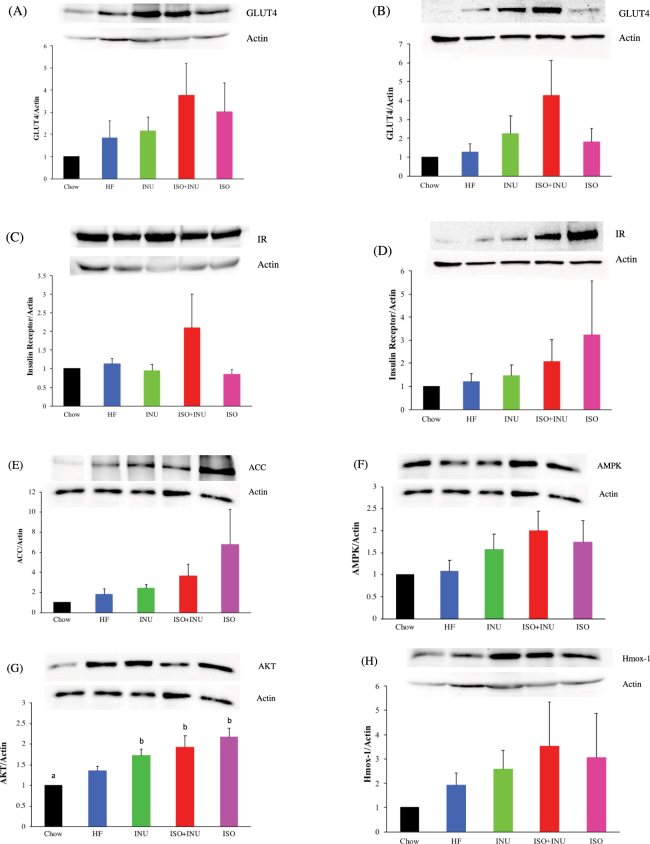
Supplementation with isoquercetin and inulin increases tissue expression of proteins involved in metabolic signalling pathways. Protein expression of GLUT4 receptor in (**A**) hepatic and (**B**) skeletal muscle tissue. Protein expression of the insulin receptor in (**C**) hepatic and (**D**) skeletal muscle tissue. Hepatic expression of (**E**) ACC (**F**) AMPK, (**G**) Akt and (**H**) Hmox-1 in mice receiving the normal diet (Chow), high fat diet (HF) or HF supplemented with isoquercetin (ISO), inulin (INU), or the combination (ISO + INU) for 11–12 weeks. Mean ± SEM, ANOVA n = 5–7 per group, different letters (a versus b) are statistically significant *p < 0.05.

Western blot analysis revealed a pronounced trend towards increased expression of the GLUT4 receptor in the liver and skeletal muscle of mice fed the HF diet supplemented with combination isoquercetin and inulin (Fig. 3A,B), which was accompanied by a trend towards increased expression of the insulin receptor in the liver and skeletal muscle (Fig. 3C,D). Hepatic ACC, AMPK and Akt expression also appeared to be increased in the mice fed a HF diet supplemented with isoquercetin either alone or in combination with inulin (Fig. 3E,G), with no effect on skeletal muscle expression (data not shown). Hepatic heme oxygenase-1 (Hmox-1) levels were elevated in all mice receiving the HF diet, although this appeared to be highest in mice receiving isoquercetin and the combination of isoquercetin and inulin (Fig. 3H).

### Isoquercetin and inulin synergistically improve the lipid profile and reduce fatty liver in mice fed a high fat diet

**Figure 4:**
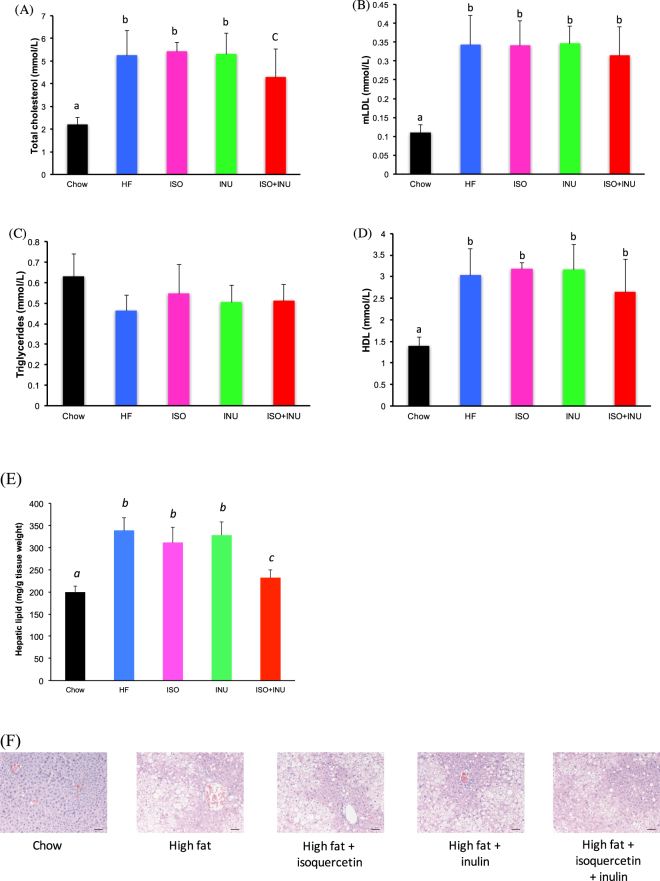
Supplementation with isoquercetin and inulin synergistically affect lipid profiles and hepatic lipid content. Fasting serum (**A**) total cholesterol, (**B**) measured low-density lipoprotein (mLDL) cholesterol, (**C**) triglyceride, and (**D**) high-density lipoprotein (HDL) cholesterol levels as well as hepatic (**E**) lipid content and (**F**) lipid droplet accumulation in mice receiving the normal diet (Chow), high fat diet (HF) or HF supplemented with isoquercetin (ISO), inulin (INU), or the combination (ISO + INU) for 12 weeks. Mean ± SEM, ANOVA n = 9–10 per group, different letters (a versus b, a versus c, b versus c) are statistically significant *p < 0.05.

Serum total cholesterol and low-density lipoprotein (LDL) levels were significantly elevated in mice fed the HF diet compared to the normal chow diet (Fig. 4A,B). The addition of isoquercetin or inulin to the diet had no effect, however the combination was able to attenuate the increase in total cholesterol (Fig. 4A). There were no differences in serum triglyceride levels between the groups (Fig. 4C) and high-density lipoprotein (HDL) was significantly elevated in all groups receiving the HF diet compared to normal chow (Fig. 4D).

Analysis of hepatic lipid content revealed significant increases in lipid accumulation in mice fed the HF diet. Supplementation with isoquercetin or inulin had no effect, while the combination significantly reduced hepatic lipid content (Fig. 4E). This finding was supported by histology, which showed an increase in lipid droplets in liver samples from mice fed the HF diet, and the HF diet supplemented with isoquercetin or inulin, compared to normal chow. Hepatic lipid droplets were reduced in mice receiving the combination diet compared to the other HF diets (Fig. 4F).

### Isoquercetin and inulin synergistically reduce adipocyte hypertrophy and alter adipokine production

**Figure 5:**
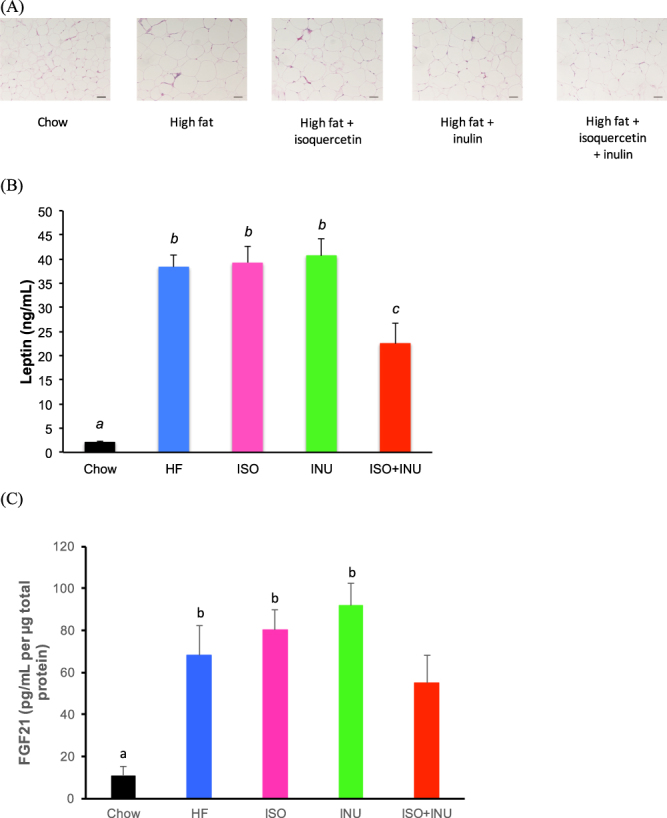
Supplementation with isoquercetin and inulin prevents adipocyte hypertrophy and adipokine levels in mice fed a high fat diet. (**A**) Adipocyte hypertrophy, (**B**) plasma leptin, and (**C**) adipocyte FGF21 concentrations in mice receiving the normal diet (Chow), high fat diet (HF) or HF supplemented with isoquercetin (ISO), inulin (INU) or the combination (ISO + INU) for 12 weeks. Mean ± SEM, ANOVA, n = 9–10 per group, different letters (a versus b, a versus c, b versus c) are statistically significant *p < 0.05.

There was observable adipocyte hypertrophy in mice fed the HF diet compared to mice fed the normal chow diet. There were no apparent effects following supplementation with either isoquercetin or inulin, however, the combination attenuated this hypertrophy (Fig. 5A). These morphological changes in adipose tissue were also accompanied by significant increases in circulating leptin levels (Fig. 5B) as well as adipose fibroblast growth factor-21 (FGF21) levels (Fig. 5C) in mice fed the HF diet and the HF diet supplemented with isoquercetin or inulin when compared to the normal chow diet. Supplementation with the combination diet significantly attenuated both circulating leptin and adipose FGF21 levels (Fig. 5B,C).

### Isoquercetin and inulin synergistically prevent diet-induced gut dysbiosis and improves gut bacteria metabolic potential

**Figure 6:**
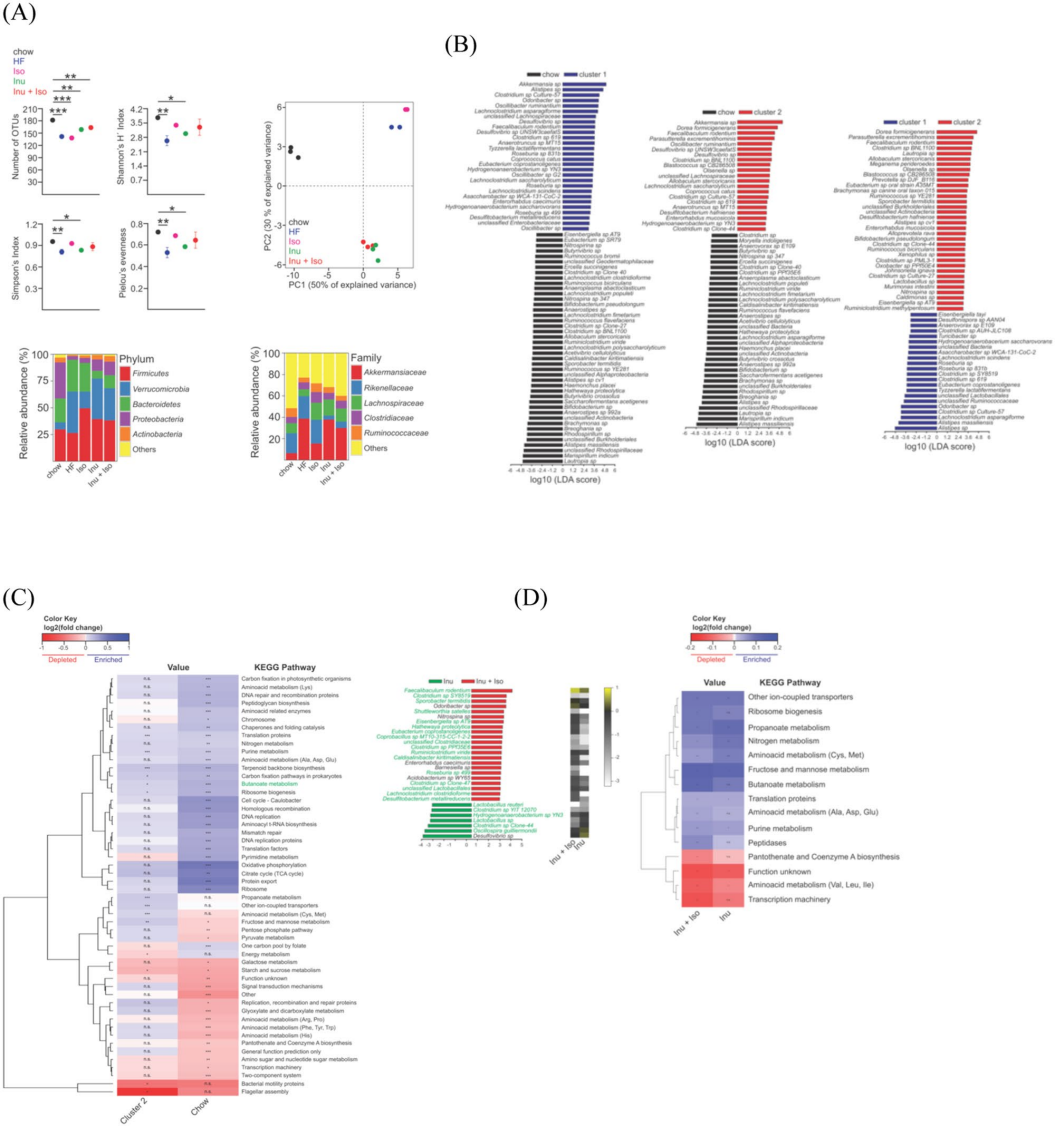
Analysis of microbiome diversity, composition and functionality in mice fed a high fat diet supplemented with isoquercetin and/or inulin. (**A**) Gut microbiome composition as determined by number of operational units (OTUs), Shannon and Simpson indices of diversity, Pielou eveness, principal component (PC) cluster analysis, and relative taxonomic composition at *Phylum* and *Family* level. (**B**) LEfSe analysis shows the combination of discriminative features that better discriminate between the indicated treatments (cluster 1 = HF diet and HF diet + ISO; and cluster 2 = HF diet + INU and HF diet + ISO + INU) versus normal chow. The LDA score (absolute value) indicates the importance of the indicated features for discriminating between treatments. (**C**) and (**D**) Heatmaps show changes in fold change (log2) in predicated metabolic capabilities of the gut microbial communities associated with the indicated treatments. For each KEGG pathway, fold changes represent the ratio between the number of genes of the indicated treatments and the number of genes of clusters (cluster 1 = HF diet and HF diet + ISO; and cluster 2 = HF diet + INU and HF diet + ISO + INU). KEGG pathways enriched or depleted after the indicated dietary interventions are shown in blue and red respectively. Dendrogram on the left groups the KEGG pathways based on the degree of similarity after hierarchical cluster analysis. All analysis performed in faecal samples collected from mice receiving the normal diet (Chow), high fat diet (HF) or HF diet supplemented with isoquercetin (ISO), inulin (INU), or the combination (ISO + INU) for 12 weeks. Statistical significance between the represented treatments was assessed by one-way ANOVA and pairwise comparisons by Tukey post hoc test. The level of significance is shown within each cell; n.s. is non-significant, *p < 0.05, **p < 0.01, ***p < 0.001.

The HF diet decreased gut bacterial diversity, as indicated by reduction of both Shannon and Simpson diversity indices. Accordingly, richness and evenness of the species distribution were also diminished by the HF diet. Supplementation with isoquercetin alone resulted in a recovery of diversity by increasing the evenness of the bacterial community, but not the number of species. Supplementation with inulin, either alone or in combination with isoquercetin, was able to moderately attenuate the reduction in richness (Fig. 6A). Principal component analysis (PCA) of microbiome composition separated the samples into three clusters, suggesting differences in gut community composition in response to the dietary intervention. The first principal component (PC1), (50% of the variance), mainly represented the variation caused by the HF diet, while PC2 (30% of the variance), represented variation due to the dietary supplementation. Interestingly, the gut microbiota of mice fed either the HF diet or HF diet with isoquercetin grouped together (cluster 1), suggesting similar composition in these communities. A similar behaviour was observed for the gut bacterial communities of mice fed with the HF diet with inulin and HF diet with isoquercetin and inulin (cluster 2) (Fig. 6A).

As expected, the HF diet affected the overall relative taxonomic composition of the faecal community, mainly impacting taxa belonging to the phyla Firmicutes, Bacteroidetes and Verrumicrobia (Fig. 6A). HF feeding resulted in a reduction of the relative proportion in members of the family Lachnospiraceae. Conversely, the proportion of gram-negative bacteria belonging to the families Akkermansiaceae (Verrumicrobia) and Rikenellaceae (Bacteroidetes) were dramatically enlarged. When isoquercetin was given alone there was an increase in the relative proportions of Firmicutes, but no reduction in the levels of Bacteroidetes (Fig. 6A). Supplementation with inulin, either alone or in combination with isoquercetin, restored the relative proportion of members within the Lachnospiraceae (Firmicutes) and reduced taxa belonging to the Rikenellaceae family (Bacteroidetes) (Fig. 6A).

Further analysis to determine the relevant OTUs, which consistently explained the differences in the gut microbiota composition between diets, revealed 71 significantly different OTUs that differentiated the normal diet cluster from cluster 1 (HF diet and HF diet with isoquercetin), 57 OTUs that differentiated the normal diet from cluster 2 (HF diet with inulin and HF diet with isoquercetin and inulin), and 56 OTUs that differentiated cluster 1 from cluster 2 (Fig. 6B).

To better understand the biological significance of the observed shifts induced by the dietary interventions in the gut bacterial community, we evaluated the metabolic potential of these communities using PICRUSt analysis. When compared to mice fed the normal chow diet, cluster 1 (HF diet and HF diet with isoquercetin) and cluster 2 (HF diet with inulin and HF diet with isoquercetin and inulin) were depleted and enriched in genes involved in similar KEGG pathways. Both cluster 1 and cluster 2 diets showed significant enrichment in KEGG pathways related to signal transduction mechanisms, transcription machinery and two-component system (data not shown). Interestingly, cluster 1 exhibited higher enrichment of genes involved in galactose metabolism, and starch and sucrose metabolism pathways, while cluster 2 was specifically enriched in genes related to propionic acid metabolism and ion-coupled transporters. Separate analysis comparing the normal chow diet and cluster 2 (HF diet with inulin and HF diet with isoquercetin and inulin) with cluster 1 (HF diet and HF diet with isoquercetin) revealed strong similarities between the metagenomic profiles of mice fed normal chow diet and the HF diet with inulin, regardless of the presence of isoquercetin. Of particular interest was the enrichment in genes related to butyric acid metabolism (Fig. 6C). Further analysis comparing the inulin containing diets revealed a higher number of OTUs specifically enriched in the HF diet with inulin and isoquercetin (22 OTUs) versus the HF diet with inulin alone (7 OTUs), with most of these being in the Firmicutes phylum (Fig. 6D). Furthermore, there was a higher number of genes related to butyric acid in the HF diet with isoquercetin and inulin, compared to HF diet with inulin (Fig. 6D), and a lower number of genes related to pantothenate metabolism, transcription machinery and branched-chain amino acid metabolism (Fig. 6D).

### Isoquercetin and inulin synergistically influence short chain fatty acid (SCFA) production

**Figure 7:**
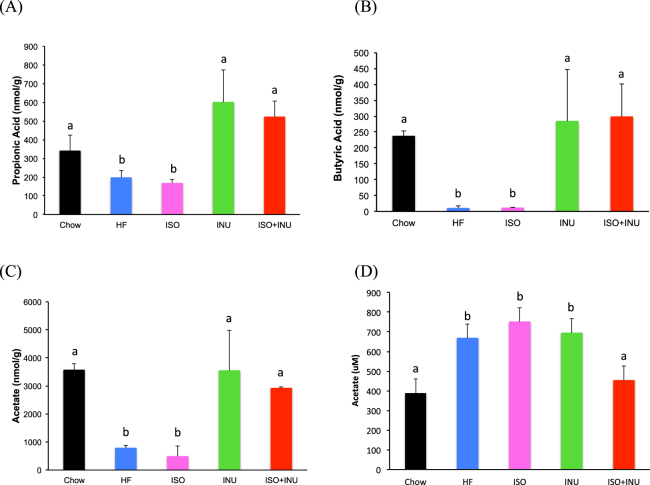
Supplementation with isoquercetin and inulin alters faecal and plasma short chain fatty acid production in mice fed a high fat diet. Concentration of (**A**) faecal propionic acid, (**B**) faecal butyric acid, (**C**) faecal acetate, and (**D**) serum acetate in mice receiving the normal diet (Chow), high fat diet (HF) or HF supplemented with isoquercetin (ISO), inulin (INU) or the combination (ISO + INU) for 12 weeks. Mean ± SEM, ANOVA, n = 5–10 per group, different letters (a versus b) are statistically significant *p < 0.05.

There were significant reductions in the faecal levels of propionic acid, butyric acid and acetate following supplementation with the HF diet compared to normal chow (Fig. 7A,C). Isoquercetin alone had no effect, while inulin, either alone or in combination with isoquercetin, significantly increased faecal levels of all three SCFAs (Fig. 7A,C). Plasma levels of propionic and butyric acid were not readily detected (data not shown), while plasma acetate levels were significantly increased with the HF diet, the HF diet with isoquercetin and the HF diet with inulin. In contrast, the combination of isoquercetin and inulin significantly reduced plasma acetate levels (Fig. 7D).

## Discussion

Population studies suggest that diets rich in fruits and vegetables help prevent metabolic diseases although it remains unclear how the specific components of such a diet act to improve health. While dietary flavonoids, a major functional component of plant-based foods, are thought to be partly responsible^12^, optimal conditions for their metabolism are important for both absorption and biological activity^5^. It’s estimated that only 5–10% of total polyphenol intake is absorbed in the small intestine, with the remaining polyphenols accumulating in the large intestinal lumen, together with conjugates excreted through the bile, where they are then subject to colonic microbiota^12^. The subsequent breakdown of these polyphenols into low molecular weight phenolic acids enhances their absorption, bioavailability and bioactivity, contributing to their direct beneficial effects^11^. Tracer studies have shown that microbiota-generated phenolic acids are more readily absorbed and often possess longer half-lives and increased systemic concentration, further facilitating their beneficial effects^29^. In addition, the phenolic acids can modulate and cause fluctuations in the microbiota through selective prebiotic effects and antimicrobial activities^12,13,29^. The beneficial effects of these phenolic acid metabolites include increased insulin-stimulated glucose uptake, energy expenditure, fatty acid oxidation and thermogenic gene expression, with reductions in inflammation, hepatic steatosis, lipogenesis, and blood pressure^29^. Dietary fibre, also common in plant-based foods, functions as a prebiotic due to its non-digestibility and ability to beneficially affect the host by selectively stimulating the growth and/or activity of bacteria in the colon^30^. In addition, fibres are broken down into SCFAs, which act as a source of energy for epithelial cells in the colon as well as modulate endogenous metabolic processes including satiety, lipolysis, insulin sensitivity and secretion, gluconeogenesis, lipid storage and AMPK activity^15,30^.

In the present study, supplementation with combination isoquercetin and inulin significantly attenuated the development of the metabolic syndrome and had a significant impact on microbiome composition and activity. This effect was so pronounced that these mice had a microbiota and metabolic profile similar to mice receiving a normal chow diet. Furthermore, when compared to the HF diet treated mice, those supplemented with isoquercetin and inulin, gained less weight despite eating the same amount of food, suggesting effects on metabolism, which was supported by normalised leptin and FGF21 levels, and enhanced hepatic AMPK, ACC and Akt expression. To our knowledge, this is the first study to describe such a finding and suggests a synergistic activity between these two dietary components. We hypothesise that this synergistic effect is being driven by two metabolically related events; (i) the inulin having a direct prebiotic effect to improve gut microbiota profiles, and (ii) this improved microbiome facilitating the absorption and metabolism of isoquercetin, thereby enhancing its direct beneficial effects. Several of our findings support this hypothesis.

Firstly, we have demonstrated that the number of OTUs specifically enriched with the combination diet is higher compared to the other HF diets, suggestive of greater functional diversity in gut microbiota. Mice receiving the combination diet also exhibited higher proportions of *Faecalibaculum rodentium*, a microorganism known to produce high quantities of lactic acid^31^. Lactate can be used by cross-feeder bacteria to produce propionate and butyrate^32^ and gut derived propionate is used in hepatic synthesis of odd-chain fatty acids, which are associated with a reduced risk of T2DM^33^. Supporting this is our observation of increased faecal propionic and butyric acid and KEGG pathway analysis revealing a higher number of genes related to butyric acid in mice receiving the combination diet. We also observed reduced plasma acetate levels with the combination diet, which is significant as gut dysbiosis that increases circulating acetate levels leads to activation of the parasympathetic nervous system and promotes insulin resistance, obesity and other features of the metabolic syndrome in rodents^34^. Furthermore, the combination diet also resulted in a reduction in the number of genes associated with the metabolism of branched-chain amino acids, which are associated with insulin resistance^35^.

Gut dysbiosis can have a significant effect on flavonoid bioavailability within the intestine, which is driven by a microbiome shift towards a flavonoid-degrading configuration^36^. This is important given some flavonoids have been shown to affect food intake, adipocyte differentiation and lipid metabolism^37,38^. Additionally, flavonoids and their metabolites have been demonstrated to have a range of beneficial effects through various signaling pathways. These include improved glucose-insulin homeostasis through increased AMPK phosphorylation, PPAR-γ and PGC-1α expression in skeletal muscle, adipose tissue and liver^29^. While food intake was not affected in the present study; hepatic lipid content, adipocyte hypertrophy, and circulating leptin levels were all beneficially influenced by the combination diet. Again, supporting a role for inulin beneficially affecting microbiome composition to enhance flavonoid bioavailability. Furthermore, hepatic and skeletal muscle expression of GLUT4, the major receptor responsible for glucose uptake^39^, and the insulin receptor were also increased with the combination diet. This was accompanied by increases in the hepatic expression of ACC, AMPK and Akt, proteins involved in both fatty acid oxidation and glucose uptake^39^.

Hmox-1 is an inducible enzyme that degrades heme to biliverdin, which is then rapidly reduced to bilirubin. Both biliverdin and bilirubin have anti-obesity and anti-glycaemic effects^40^, while increased Hmox-1 levels ameliorate fatty liver development through recruitment of FGF21^41^. Hmox-1 can also inhibit pre-adipocyte proliferation and differentiation via activation of Akt^42^ and FGF21 has been demonstrated to be a regulator of glucose and lipid homeostasis, with FGF21 levels abnormally elevated in insulin resistant states^43^. We have previously demonstrated that Hmox-1 plays a critical role in the vascular protection activity of quercetin^44–46^. In the present study, the combination of isoquercetin and inulin attenuated both weight gain and adipocyte hypertrophy. This was accompanied by significant reductions in adipose levels of FGF21 and plasma levels of leptin, which may be indicative of a normalised metabolic profile. Accompanying this were increases in the hepatic expression of both Akt and Hmox-1.

Bacterial composition and diversity in the gut is considered an indicator of overall health status^6,7,9^. Supporting this, we observed a significant reduction in bacterial diversity in mice fed a HF diet compared to normal chow. Specifically, the HF diet increased taxa belonging to the phyla Bacteroidetes and Verrucomicrobia. This increase in gram-negative bacteria is in agreement with previous reports where HF feeding results in gut microbiota composition with increased capacity to produce LPS, which has been demonstrated to trigger obesity and diabetes^47^. We also observed a decrease in taxa belonging to the phylum Firmicutes, mainly within the Lachnospiraceae family, which synthesise butyric acid^48^, along with profound reductions in faecal butyric acid levels. In addition to these microbiome changes, we observed the development of several features of the metabolic syndrome, including obesity, glucose intolerance, insulin resistance, fatty liver, adipocyte hypertrophy and increased circulating leptin. This strongly supports an association between gut dysbiosis and health status.

Supplementation of the HF diet with isoquercetin had no effect on any of the metabolic parameters, suggesting no direct beneficial effects of this dietary flavonoid when given with the HF diet. This is in contrast to a recent study which showed the same dose of quercetin was able to reduce obesity-induced hepatosteatosis in mice fed a HF diet (comprised of 60% fat) for 9 weeks^49^. Interestingly in our study, the isoquercetin supplemented HF diet clustered with the HF diet, suggesting a similarity in gut composition, which may help explain the lack of benefit we observed. However, the isoquercetin was able to restore some diversity and evenness in the gut community, supporting a role for some limited prebiotic effects. When mice receiving the HF diet were supplemented with inulin, there was also no significant effect on development of the metabolic syndrome, despite improvements in microbiome composition and SCFA levels. This is not unexpected given inulin functions as a prebiotic, which is broken down by gut bacteria to produce SCFAs^50^. The lack of benefit on features of the metabolic syndrome however, is in contrast to two recent studies. In the first study, inulin was able to prevent diet-induced adiposity in mice fed a HF diet, which was microbiota and SCFA dependent^10^. More recently, inulin has been demonstrated to protect against HF diet-induced metabolic syndrome by nourishing microbiota to restore interlukin-22 mediated enterocyte function, which was not SCFA dependent^51^. While we failed to show any protective effect of inulin against development of the metabolic syndrome, despite changes to the microbiome and SCFA production, the discrepancies between our study and the two studies carried out by Gewirtz *et al*. are likely due to differences in diet composition, including inulin dose, food intake and treatment times. Worth noting is that the inulin dose used in our study is physiologically relevant and when converted to human doses^18^ is equivalent to the recommended daily intake of fibre (25–30 g).

While isoquercetin bioavailability (plasma or tissue levels) to confirm the role of inulin in enhancing its metabolism and absorption has not specifically been investigated in the present study, the profound effect the combination had in attenuating development of the metabolic syndrome compared to when isoquercetin was given alone, supports this hypothesis. Furthermore, our results demonstrate how different dietary regimes can influence host bacterial composition and improve metabolic profiles, suggestive of an association between the gut’s bacterial community and disease status. Taken together, these results suggest that inulin beneficially changes gut microbiome composition through its prebiotic effects and this shift enhances the metabolism and absorption of isoquercetin, facilitating its direct beneficial effects and supporting a synergistic mode of action between these two important dietary components. Our findings are underpinned by a recent review, which has highlighted the cardiometabolic health benefits of microbiota-generated flavonoid metabolites, supporting a bidirectional interaction between dietary components and gut microbiota with host health^29^.

In conclusion, we have demonstrated that long-term supplementation with the dietary flavonoid isoquercetin and the soluble fibre inulin can prevent development of the metabolic syndrome in mice fed a high fat diet. This protective effect appears to be mediated through beneficial changes to the microbiome, enhanced flavonoid bioavailability and bioactivity, and direct effects of both inulin and isoquercetin. Together, these findings highlight the important role diet plays in the prevention of cardiometabolic diseases and enhances our understanding of the role of individual dietary components.
